# Homoharringtonine Attenuates Dextran Sulfate Sodium-Induced Colitis by Inhibiting NF-*κ*B Signaling

**DOI:** 10.1155/2022/3441357

**Published:** 2022-09-29

**Authors:** Jiangrui Liu, Liangpan Shi, Wenchang Huang, Zhihua Zheng, Xiaohui Huang, Yibin Su

**Affiliations:** Department of Gastrointestinal Surgery, Quanzhou First Hospital Affiliated to Fujian Medical University, No. 248 Dong Street, Licheng District, Quanzhou, 362000 Fujian, China

## Abstract

Homoharringtonine (HHT) exhibits an anti-inflammatory activity. The potential protective effects and mechanisms of HHT on dextran sulfate sodium- (DSS-) induced colitis were investigated. DSS-induced colitis mice were intraperitoneally injected with HHT. Body weight, colon length, disease activity index (DAI), and histopathological change were examined. The relative contents of interleukin- (IL-) 1*β*, tumor necrosis factor- (TNF-) *α*, IL-6, and the chemokine (C-C motif) ligand 2 (CCL2) in the colon tissues and HHT-treated RAW264.7 cells were detected with the enzyme-linked immunosorbent assay. In the meantime, the levels of p-p65 and p-I*κ*B*α* were detected by Western blot. The proportion of macrophages (CD11b^+^F4/80^+^) in the colon tissues was detected by flow cytometry. HHT alleviated DSS-induced colitis with downregulated TNF-*α*, IL-1*β*, IL-6, and CCL2 expression; reduced activation of nuclear factor-kappa B (NF-*κ*B) signaling; and diminished proportion of recruited macrophages in colon tissues. It was further testified that HHT inhibited lipopolysaccharide-induced macrophage activation with reduced activation of NF-*κ*B signaling. In addition, HHT inhibited the M1 polarization of both human and mouse macrophages, while HHT did not affect the differentiation of human CD4 T cells into Th17, Th1, or Treg cells and did not affect the proliferation and migration of human colon epithelial cells. In summary, HHT attenuates DSS-induced colitis by inhibiting macrophage-associated NF-*κ*B activation and M1 polarization, which could be an option for the treatment of ulcerative colitis.

## 1. Introduction

Characterized by the abnormal inflammation in the inner surface of the rectum and colon, ulcerative colitis (UC) demonstrates relapsing and remitting symptoms. The prevalence of UC ranges from 1% to 2% [1]. Immune reactions medicated by dendritic cells, macrophages, and T cells may contribute to the development of UC [2–4]. 5-Aminosalicylic acid (5-ASA) drugs, corticosteroids, immune suppressants, and biological drugs are recommended to treat human UC, while due to the limited efficacy and safety consideration for long-term medication, there is an increasing demand to develop a viable solution [5].

Dextran sulfate sodium- (DSS-) induced colitis is extensively utilized to decipher the contribution of innate immune reactions to the development of UC [6–8]. Previous studies demonstrate that intestinal macrophages drive DSS-induced colitis, which can release proinflammatory cytokines and chemokines to destroy the intestinal barrier and lead to tissue damage [9, 10]. These findings indicate that targeted therapy against macrophages might be a treatment option.

As a plant cephalotaxine alkaloid derived from *Cephalotaxus*, homoharringtonine (HHT) can prevent the initial elongation step of protein synthesis to halt protein translation. It is also reported that HHT can reversibly inhibit antiapoptotic protein expression and reduce IL-6-induced STAT3 phosphorylation [11]. Recently, HHT has been testified to have antitumor properties to treat chronic myeloid leukemia (CML) as recommended by the US Food and Drug Administration [12]. Some studies have also shown that HHT can regulate nuclear factor-kappa B (NF-*κ*B) to inhibit allergic inflammation [13] and demonstrate broadly *in vitro* and *in vivo* antiviral activity [14, 15].

## 2. Methods and Materials

### 2.1. DSS-Induced Colitis

Pathogen-free grade male C57BL/6 mice (8-week-old, 20 ± 2 g) were obtained from Vital River Laboratory Animal Ltd. (Beijing, China) and received 3% DSS (MP Biomedicals, USA) in autoclaved drinking water for seven days. Based on weight monitoring, bleeding, and stool consistency, the disease activity index (DAI) was evaluated as previously reported [16]. All experimental procedures were approved by the Animal Ethics Committee of Quanzhou First Hospital affiliated to Fujian Medical University. HHT was ordered from Selleck, which was further intraperitoneally (i.p.) injected for 7 days (1 mg/kg or 2 mg/kg).

### 2.2. RAW264.7 Macrophage Cells

The RAW264.7 macrophage cells were ordered from China Center for Type Culture Collection and cultured in Dulbecco's Modified Eagle's medium (DMEM) supplemented with 10% fetal bovine serum (FBS, HyClone), which was further treated with 1 *μ*g/mL lipopolysaccharide (LPS) along with vehicle or 50 nM HHT for 6 h.

### 2.3. Macrophage Polarization

C57BL/6 mice were i.p. injected with 3% Brewer thioglycollate medium, and peritoneal macrophages were elucidated and cultured in complete DMEM (supplemented with 10% FBS) at 37°C with 5% CO_2_. In order to induce M1 macrophages, 100 ng/mL LPS and 20 ng/mL interferon- (IFN-) *γ* (PeproTech) were added and incubated for 2 h. In order to induce M2 macrophages, 50 ng/mL recombinant mouse IL4 (R&D Systems) was added and incubated for 24 h. After 15 minutes' incubation, the induced M1 or M2 macrophages were harvested for further Western blot and RT-PCR analysis.

### 2.4. Single-Cell Preparation and Flow Cytometry Analysis

Mesenteric lymph nodes and spleens were smashed and filtered through 40 *μ*m nylon mesh strainers to get single-cell suspensions. Colon fragments were shaken and incubated with Mg2^+^-free Hanks' buffer saline containing 5 mmol/L EDTA for 1 h, which was then enzyme digested with 1 mg/mL collagenase and 25 *μ*g/mL DNase in RPMI-1640 medium. PE-anti-F4/80 and PercP-Cy5.5-anti-CD11b antibodies were utilized to stain macrophages. Flow cytometry analysis was performed on BD FACSCanto II Flow Cytometry System.

### 2.5. Isolation of Human Macrophages and T Cells

MACSprep™ PBMC Isolation Kit (Miltenyi Biotec) was utilized to extract peripheral blood mononuclear cells (PBMC) from healthy control people or UC patients, which were further loaded onto CD14 MicroBeads (Miltenyi Biotec) to obtain monocytes according to the manufacturer's instructions. The monocytes (10^6^ cells/mL) were stimulated with 100 ng/mL macrophage colony-stimulating factor (M-CSF; PeproTech) for seven days to induce the differentiation of macrophages. Then, 100 ng/mL LPS and 20 ng/mL IFN-*γ* (PeproTech) were added and incubated along with vehicle or 50 nM HHT for 2 h to induce the differentiation of M1 macrophages or 50 ng/mL IL4 was added and incubated along with vehicle or 50 nM HHT for 24 h to induce the differentiation of M2 macrophages.

Human CD4^+^ T Cell Isolation Kit (Miltenyi Biotec) was utilized to purify CD4^+^T from PBMC, which were stimulated with plate-bound 5 *μ*g/mL anti-CD3 and 2 *μ*g/mL anti-CD28 for 72 h in RPMI-1640 medium (10% FBS, 1 mM sodium pyruvate, 10 mM HEPES, and 50 *μ*M 2-mercaptoethanol). All participants provided written consent, and this study were reviewed and approved by the Ethical Committee of Fujian Medical University.

### 2.6. Wound Healing Assay

FHC cells obtained from ATCC were planted (5 × 10^6^) into six-well plates and incubated with DMEM supplemented with 10% FBS. When the FHC cells reached 90% of confluence, a 200 *μ*L sterile tip was utilized to scratch the cell monolayer.

### 2.7. RT-qPCR

RNAiso Plus (Takara) was used to extract total cellular RNA, which was reverse-transcribed into cDNA with M-MLV transcriptase (Promega). Quantitative RT-PCR was performed on an ABI StepOne PCR System (Applied Biosystems) with SYBR Green Master Mix (DBI Bioscience), with an initial denaturation of 95°C for 10 min, and followed by 40 cycles of 95°C for 15 s and 60°C for 1 min. GAPDH was used as an internal control. Primer sequences are listed in Table S1.

### 2.8. Western Blot

Cultured RAW264.7 macrophages cells and colon tissues were homogenated and lysed with radioimmunoprecipitation lysis buffer (Beyotime, China), which was further centrifuged (4°C, 12,000 × g, 15 min) to collect the supernatant. The supernatant (30 *μ*g) was then separated with 12% sodium dodecyl sulfate–polyacrylamide gel electrophoresis and transferred to nitrocellulose membranes. The blots were blocked with 5% (*w*/*v*) skim milk and incubated with primary antibodies against GAPDH, p-I*κ*B*α*, p-p65, p65, p-p38, p-JNK, p-AKT, and p-ERK (4°C, overnight), which were further incubated with HRP-conjugated secondary antibodies (room temperature, 1 h). Finally, the density of interest protein was normalized to GAPDH with ImageJ software.

### 2.9. Enzyme-Linked Immunosorbent Assay (ELISA)

The concentration of IL-1*β*, TNF-*α*, IL-6, and CCL2 in the lysed cultured RAW264.7 macrophages and colon tissue supernatant was detected with DuoSet ELISA kits (R&D Systems) according to the manufacturer's suggestion. The absorbance was measured at 450 nm in the SpectraMax M5 microplate reader (Molecular Devices).

### 2.10. Statistical Analysis

The significant difference was analyzed using two-tailed *t*-test, one-way ANOVA, or two-way ANOVA test with Tukey's multiple-comparison test in GraphPad prism 7. Error bar represents mean ± SD. It was considered statistically significant when *p* < 0.05.

## 3. Results

### 3.1. HHT Alleviates DSS-Induced Colitis in Mice

**Figure 1:**
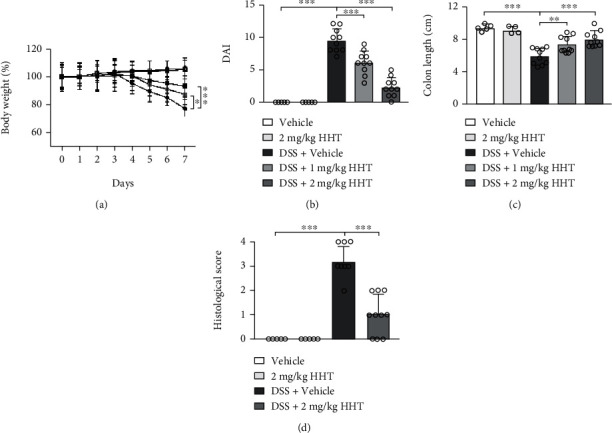
Effect of HHT on DSS-induced colitis in mice. Mice were treated with 3% DSS in autoclaved drinking water along with intraperitoneally injected vehicle, 1 mg/kg HHT, or 2 mg/kg HHT for seven days: (a) bodyweight of mice; (b) DAI (disease activity index) on day 7; (c) colon length or mice on day 7; (d) histopathological scores from colon tissue sections of DSS-induced mice on day 7. Five mice per group. Error bar represents mean ± SD. ^∗^*p* < 0.01, ^∗∗^*p* < 0.01, and ^∗∗∗^*p* < 0.001.

DSS-exposed mice showed dramatic body weight reduction from day 4 (Figure 1(a)) with increased DAI (Figure 1(b)), colon shortening (Figures 1(c) and 1(d)), and pathological inflammatory cell infiltration (Figures 1(e) and 1(f)), which indicated the success of DSS induced-colitis model construction. Both 1 mg/kg HHT and 2 mg/kg HHT could restore the alteration of body weight (Figure 1(a)), DAI (Figure 1(b)), colon length (Figure 1(c)), and colon pathology (Figure 1(d)) of DSS-induced colitis mice. It was worth noting that none of the vehicle-treated healthy control mice or HHT alone-treated healthy control mice exhibited pathological changes, indicating the safety of HHT treatment. These data testify that HHT could alleviate DSS-induced colitis.

### 3.2. HHT Inhibits DSS-Induced Inflammation in the Colon

**Figure 2:**
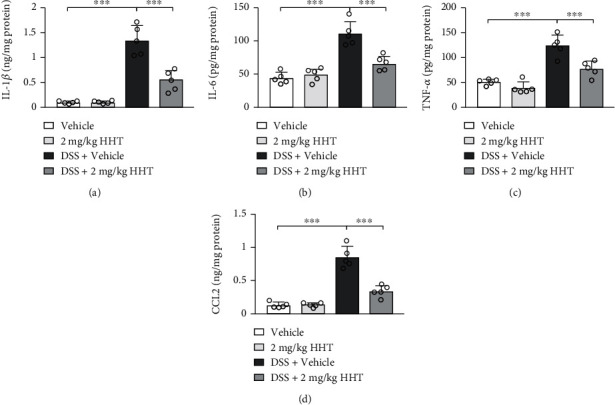
HHT inhibits DSS-induced colon inflammation. Mice were treated with 3% DSS along with vehicle or 2 mg/kg HHT for seven days, and the colon tissues were homogenized for ELISA of IL-1*β* (a), IL-6 (b), TNF-*α* (c), and CCL2 (d). Five mice per group. Error bar represents mean ± SD. ^∗∗∗^*p* < 0.001.

To decipher the relevant mechanism mediated by HHT, proinflammatory cytokines in the colon were detected with ELISA. DSS alone could induce remarkable elevation of IL-1*β* (Figure 2(a)), IL-6 (Figure 2(b)), TNF-*α* (Figure 2(c)), and CCL2 (Figure 2(d)) expression compared with the vehicle-treated mice, which could be reversed by HHT administration. However, the expression of such proinflammatory cytokines was not affected by HHT administration in healthy control mice compared with vehicle-treated healthy mice. All of these indicate that the alleviation of DSS-induced colitis might be attributed to the inhibition of proinflammatory cytokine expression.

### 3.3. HHT Inhibits DSS-Induced Macrophage Recruitment in the Colon

**Figure 3:**
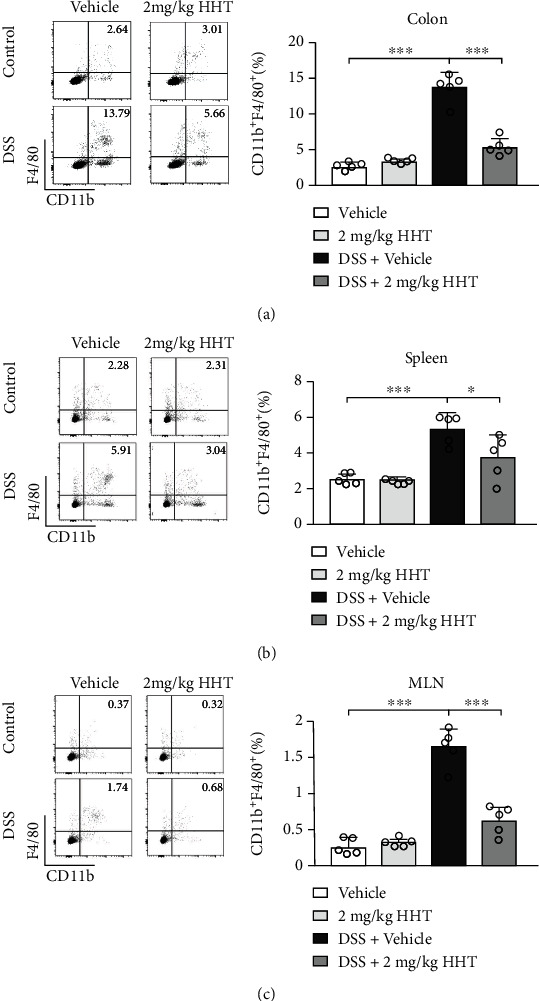
HHT inhibits the recruitment of macrophages induced by DSS in the colon. Mice were treated with 3% DSS along with vehicle or 2 mg/kg HHT for seven days, and the colon tissues (a), spleens (b), and mesenteric lymph nodes (MLN) (c) were harvested for FACS analysis. The proportion of macrophages (CD11b^+^F4/80^+^ cells) was determined. Five mice per group. Error bar represents mean ± SD. ^∗^*p* < 0.05; ^∗∗∗^*p* < 0.001.

The proportion of macrophages in the colon, spleen, and mesenteric lymph nodes was determined in DSS-induced colitis to evaluate the dynamic changes of macrophage (CD11b^+^F4/80^+^) traffic. As expected, HHT treatment did not alter the proportion of macrophages in the healthy mice compared with the vehicle-treated mice. In comparison, DSS-induced colitis mice showed upregulated macrophage enrichment in the colon (Figure 3(a)), spleen (Figure 3(b)), and mesenteric lymph nodes (Figure 3(c)), which could be reversed by HHT treatment. The treatment benefit of HHT might be attributed to the diminished infiltration of macrophages in the colon.

### 3.4. HHT Inhibits the Activation of Macrophages Induced by LPS *In Vitro*

**Figure 4:**
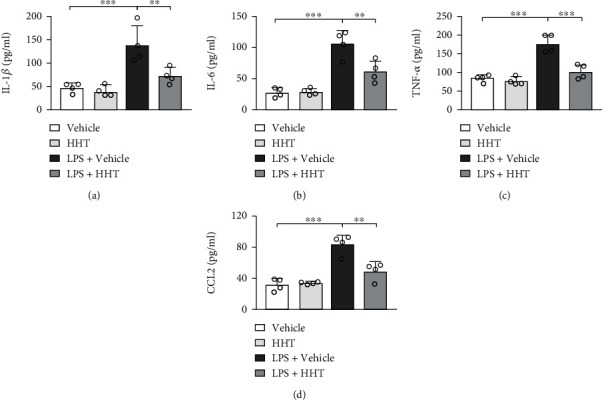
HHT inhibits the activation of macrophages induced by LPS *in vitro*. Cultured RAW264.7 cells were stimulated with 1 *μ*g/mL LPS along with vehicle or 50 nM HHT for 6 hours. And the medium was harvested for the ELISA of IL-1*β* (a), IL-6 (b), TNF-*α* (c), and CCL2 (d). Error bar represents mean ± SD. ^∗∗^*p* < 0.01; ^∗∗∗^*p* < 0.001.

LPS was utilized to stimulate the activation of RAW264.7 cells with the upregulated proinflammatory cytokine expression, such as IL-1*β* (Figure 4(a)), IL-6 (Figure 4(b)), TNF-*α* (Figure 4(c)), and CCL2 (Figure 4(d)), which could be downregulated by HHT treatment. In comparison, HHT alone did not alter the relative content of proinflammatory cytokine induction as detected by ELISA in the supernatant of RAW264.7 cells compared with the vehicle-treated group. These results indicate that the downregulation of proinflammatory cytokines in DSS-induced colitis mice could be attributed to the inhibition of macrophage activation mediated by HHT.

### 3.5. HHT Reduces the Activation of NF-*κ*B Signaling *In Vitro* and *In Vivo*

**Figure 5:**
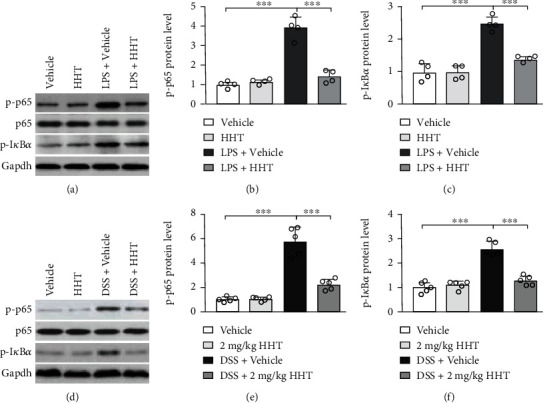
HHT reduces the activation of NF*κ*B signaling *in vitro* and *in vivo*. (a–c) Cultured RAW264.7 cells were stimulated with 1 *μ*g/mL LPS along with vehicle or 50 nM HHT for 1 hour. The level of p-p65 (a, b), p65 (a), and p-I*κ*B*α* (a, c) was analyzed by Western blot. (d–f) Mice were treated with 3% DSS along with vehicle or 2 mg/kg HHT for seven days, and the colon tissues were harvested to determine the protein level of p-p65 (d, e) and p-I*κ*B*α* (d, f) by Western blot. Five mice per group. Error bar represents mean ± SD. ^∗∗∗^*p* < 0.001.

A significant upregulation of p-p65 (Figures 5(a) and 5(b)) and p-I*κ*B*α* (Figures 5(a) and 5(c)) expression was observed in RAW264.7 macrophage cells after LPS stimulation, which could be decreased by HHT administration. Meanwhile, a significant upregulation of p-p65 (Figures 5(d) and 5(e)) and p-I*κ*B*α* (Figures 5(d) and 5(f)) was also observed in the colon of DSS-induced colitis, which was significantly downregulated after HHT treatment. Thus, it is concluded that the downregulated NF-*κ*B signaling contributes to the diminished proinflammatory cytokine expression in macrophages and the alleviation of colitis pathology.

### 3.6. HHT Inhibits the M1 Polarization of Mouse Macrophages

**Figure 6:**
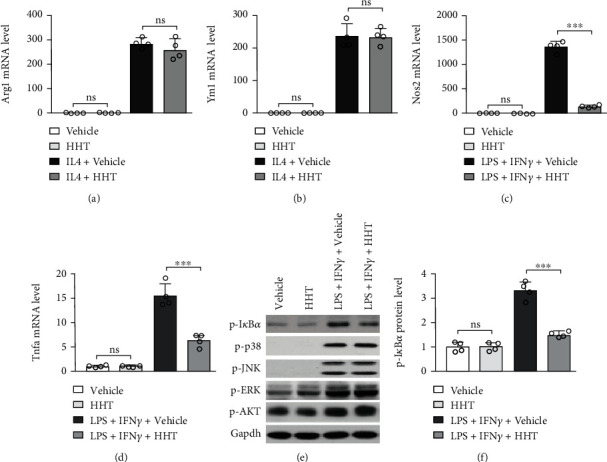
HHT inhibits the M1 polarization of macrophages. (a, b) Peritoneal macrophages were incubated with 50 ng/mL recombinant mouse IL4 along with vehicle or 50 nM HHT for 24 hours. The mRNA level of Arg1 (a) and Ym1 (b) was analyzed by RT-qPCR. (c, d) Peritoneal macrophages were treated with 100 ng/mL LPS and 20 ng/mL IFN*γ* along with vehicle or 50 nM HHT for 2 hours. The mRNA level of Nos2 (c) and TNF-*α* (d) were analyzed by RT-qPCR. (e, f) Peritoneal macrophages were stimulated with 100 ng/mL LPS and 20 ng/mL IFN*γ* along with vehicle or 50 nM HHT for 15 min. The protein level of p-I*κ*B*α* (e, f), p-AKT, p-JNK, p-p38, and p-ERK (e) was analyzed by Western blot. Error bar represents mean ± SD. ^∗∗∗^*p* < 0.001; ns: not significant.

The effect of HHT on macrophage polarization was further investigated. IL-4 stimulation could induce the differentiation of M2 macrophages, as indicated by the upregulated expression of Arg1 (Figure 6(a)) and Ym1 (Figure 6(b)). HHT did not affect the relative expression of Arg1 and Ym1, which indicated that HHT did not affect the polarization of M2 macrophage. On the other hand, as expected, LPS and IFN*γ* stimulation could promote the differentiation of M1 macrophages as indicated by upregulated Nos2 (Figure 6(c)) and TNF-*α* (Figure 6(d)) expression, and the upregulated Nos2 and TNF-*α* expression could be reversed by HHT. Western blot analysis demonstrated that LPS and IFN*γ* stimulation could upregulate the relative expression of p-JNK, p-p38, p-AKT, p-ERK, and p-I*κ*B*α* (Figure 6(e)), while HHT could significantly downregulate the relative expression of p-I*κ*B*α* (Figure 6(f)). To our surprise, AKT and mitogen-activated protein kinases (MAPK), such as JNK, p38, and ERK, were not affected by the administration of HHT. Therefore, HHT could inhibit the differentiation of M1 macrophages by inhibiting the activation of NF*κ*B.

### 3.7. HHT Inhibits the M1 Polarization of Human Macrophages

It was worth noting that HHT did not affect the differentiation of human M2 macrophages but inhibited the differentiation of human M1 macrophages as indicated by no affection on ARG1 (Figure S1A) and CHI3L1 (Figure S1B) expression, but significant inhibition of Nos2 (Figure S1C) and TNF (Figure S1D) expression. On the other hand, CD^+^4 T cells derived from healthy control or ulcerative colitis patients were activated by anti-CD3 and anti-CD28 antibodies along with vehicle or 50 nM HHT for 72 hours. Anti-CD3 and anti-CD28 antibody incubation could induce the relative expression of IL17A (Figure S2A), RORC (Figure S2B), TNF (Figure S2C), IFN-*γ* (Figure S2D), T-bet (Figure S2E), and FOXP3 (Figure S2F). In comparison, the addition of HHT did not affect the relative expression of such molecules, which demonstrated that HHT administration did not affect the differentiation of CD4^+^T cells into Th17, Th1, or Treg cells *in vitro*. Proliferation and wound healing assay testified that HHT did not affect the proliferation (Figure S3A) and migration (Figure S3B) ability of human colon epithelial cells *in vitro*.

## 4. Discussion

In this investigation, we demonstrate the treatment benefit of HHT in DSS-induced colitis and clarify the underlying NF-*κ*B signaling mediated proinflammatory cytokines secretion, chemokines expression, macrophage enrichment, and M1 polarization mechanism. It is worth noting that HHT inhibits the M1 polarization of both mouse and human macrophages. On the other hand, HHT does not affect the differentiation of human CD^+^4 T cells into Th17, Th1, or Treg cells and does not affect the proliferation and wound healing ability of human colon epithelial cells. HHT has been widely utilized in leukemia treatment in China for more than 30 years due to its low price and high efficiency [17]. Considering the specific target effect on macrophages, this investigation indicates the potential of HHT to treat human ulcerative colitis.

Although the exact etiology of ulcerative colitis is not well known, excessive NF-*κ*B activation has been detected in both human ulcerative colitis and murine DSS-induced colitis [18, 19], and abnormal NF-*κ*B activation has been considered as the primary mechanism that contributed to the development and progression of ulcerative colitis [20, 21]. Thus, NF-*κ*B inhibition can be considered as a treatment strategy for ulcerative colitis management.

In principle, HHT can bind to NF-*κ*B repressing factor (NKRF) and shift NKRF from the nucleus to the cytoplasm to strengthen the interaction between p65 and NKRF, which can interfere with the formation of the p65-p50 complex and thereby attenuate the transactivation activity of p65 on proinflammatory cytokines [13, 22]. These reports indicate that HHT-induced alleviation of DSS-induced colitis may be attributed to the binding of HHT with NKRF to inhibit the NF-*κ*B-mediated inflammation process.

NF-*κ*B signaling activation contributes to the M1 macrophage polarization and the subsequent proinflammatory effects upon mechanical stretch or inflammation stimulation [23, 24]. In this study, we testify that HHT could inhibit M1 macrophage polarization by inhibiting NF-*κ*B signaling in colitis. It is worth noting that HHT does not inhibit AKT and MAPK signals in macrophages. The association of NF-*κ*B with M1 polarization is also testified in previous research. Loganin could inhibit M1 polarization and modulate the sirt1/NF-*κ*B signaling to attenuate ulcerative colitis [25], and dopamine receptor D5 signaling could downregulate NF-*κ*B signaling to modulate colonic M1/M2 macrophage polarization in colitis [26].

Macrophage polarization plays a vital role in the development and remission of DSS-induced colitis, which is associated with individual responsiveness to pharmacology medication in clinical practice [27, 28]. It is worth noting that RA-specific autoantibodies to citrullinated proteins complexed with fibrinogen could stimulate macrophage activation and the release of TNF-*α* through Fcgamma receptor IIa engagement [29], which indicates that the concentration of RA-specific autoantibodies might be associated with macrophage activation. Thus, it is essential to define macrophage phenotype and autoantibody content to select responsive patients for HHT therapy. It is worth noting that the NF-*κ*B signal pathway may also mediate homeostatic function in the gut under basal conditions, even in DSS-induced colitis [30, 31]. Accordingly, total deletion of IKK*γ* in gut epithelial cells [32] and pharmacological IKK-*β* inhibition result in pronounced DSS-induced inflammation [33]. All of these indicate the importance of precise NF-*κ*B targeting in human ulcerative colitis.

## 5. Conclusion

In conclusion, this study demonstrates that HHT could suppress macrophage M1 polarization and inflammatory cytokine expression through the NF-*κ*B signaling pathway. When considering the wide application and safety in tumor treatment, HHT may potentially be a promising medicine for treating human ulcerative colitis.

## Data Availability

The data used to support the findings in this study are available from the corresponding author upon reasonable request.

## Supplementary Materials

Supplementary Materials Supplementary figures include Figures S1, S2, and S3.
