# Supplementary material for: Homoharringtonine Attenuates Dextran Sulfate Sodium-Induced Colitis by Inhibiting NF-κB Signaling
Source: Mediators Inflamm. 2022 Sep 29;2022:3441357. doi: 10.1155/2022/3441357 (PMC9536985; doi:10.1155/2022/3441357)
Supplement: Supplementary Materials — Supplementary figures include Figures S1, S2, and S3. [file 3441357.f1.docx]

**Supplementary materials**


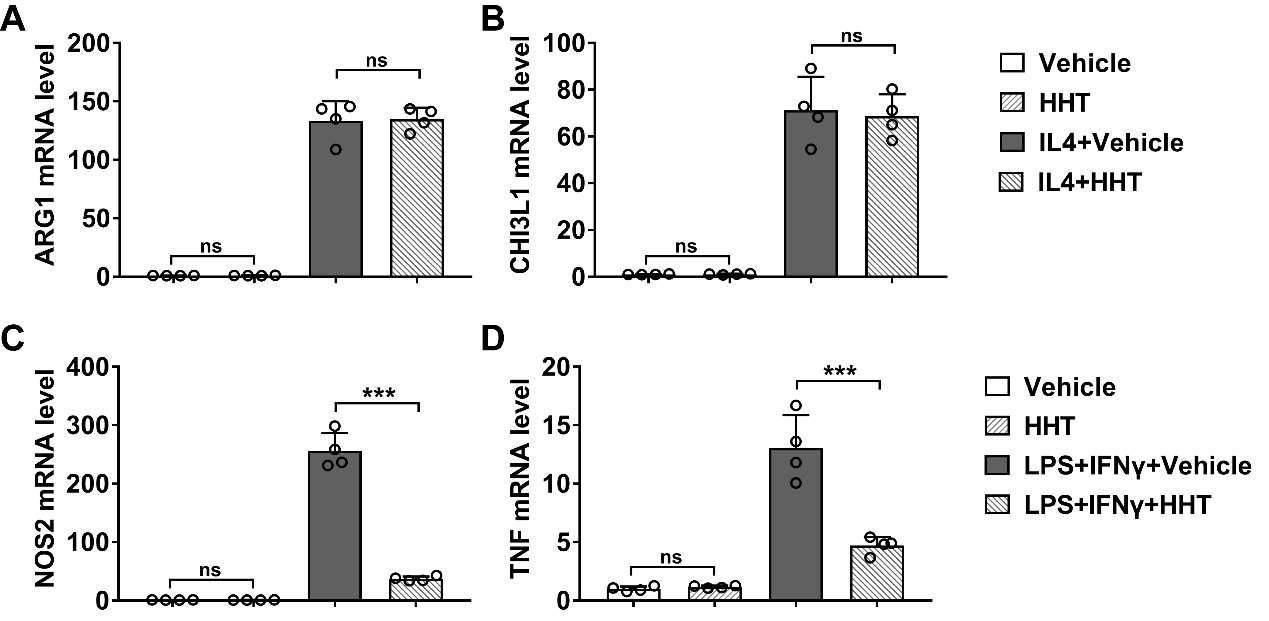


**Figure S1. HHT inhibits M1 polarization of human macrophages.**

(A-B) Cultured human macrophages from healthy donor were treated with 50 ng/mL recombinant human IL4 along with vehicle or 50 nM HHT for 24 hours. The mRNA level of ARG1 (A) and CHI3L1 (B) were analyzed by RT-qPCR. (C-D) Cultured human macrophages were treated with 100 ng/mL LPS and 20 ng/mL human IFNγ along with vehicle or 50 nM HHT for 2 hours. The mRNA level of NOS2 (C) and TNF (D) were analyzed by RT-qPCR. n=4. Error bar represents mean ± SD. ****p*<0.001, ns, not significant.


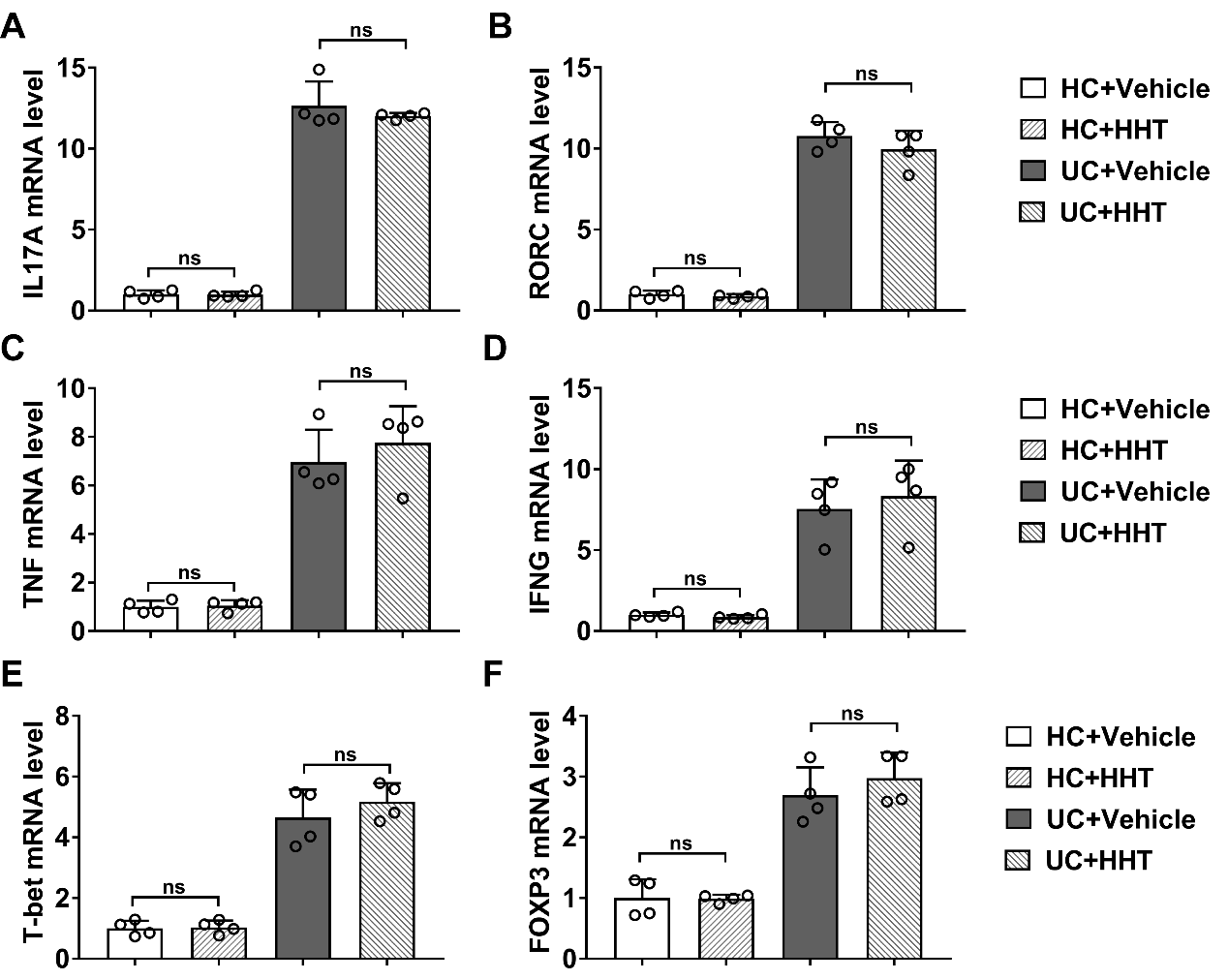


**Figure S2. HHT did not influence the differentiation of CD4 T cells from healthy people or ulcerative colitis patients *in vitro*.**

CD4 T cells from healthy control people (HC) or ulcerative colitis (UC) patients were activated by anti-CD3 and anti-CD28 along with vehicle or 50 nM HHT for 72 hours. The mRNA level of IL17A (A), RORC (B), TNF (C), IFNG (D), T-bet (E), and FOXP3 (F) were analyzed by RT-qPCR. n=4. Error bar represents mean ± SD. ns, not significant.


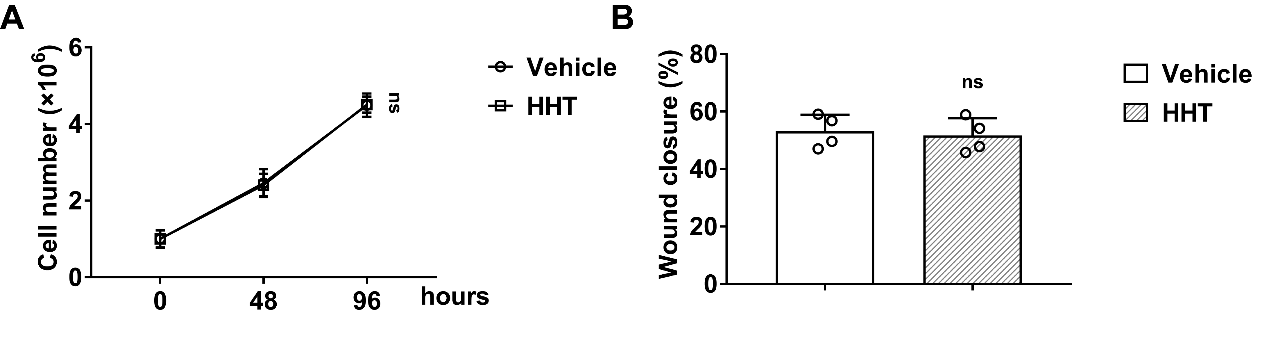


**Figure S3. HHT did not influence the proliferation and migration human colon epithelial cells *in vitro*.**

(A) Cell number of FHC cells treated with vehicle or 50 nM HHT along time. (B) Wound closure rate of FHC cells treated with vehicle or 50 nM HHT in the wound healing assay. n=4. Error bar represents mean ± SD. ns, not significant.

**Table S1**. Primer used in the PCR experiments.

| **Gene** | **Forward (5’-3’)** | **Reverse (5’-3’)** |
| --- | --- | --- |
| Gapdh | TGTAGACCATGTAGTTGAGGTCA | AGGTCGGTGTGAACGGATTTG |
| Arg 1 | CTCCAAGCCAAAGTCCTTAGAG | GGAGCTGTCATTAGGGACATCA |
| Ym1 | CAGGTCTGGCAATTCTTCTGAA | GTCTTGCTCATGTGTGTAAGTGA |
| Nos2 | GGAGTGACGGCAAACATGACT | TCGATGCACAACTGGGTGAAC |
| Tnfα | CCAAGGCGCCACATCTCCCT | GCTTTCTGTGCTCATGGTGT |
| CHI3L1 | GTGAAGGCGTCTCAAACAGG | GAAGCGGTCAAGGGCATCT-3 |
| GAPDH | CTGACTTCAACAGCGACACC | TGCTGTAGCCAAATTCGTTGT |
| ARG 1 | GTGGAAACTTGCATGGACAAC | AATCCTGGCACATCGGGAATC |
| NOS2 | TTCAGTATCACAACCTCAGCAAG | TGGACCTGCAAGTTAAAATCCC |
| TNFα | CCTCTCTCTAATCAGCCCTCTG | GAGGACCTGGGAGTAGATGAG |
| IL17A | TCCCACGAAATCCAGGATGC | GGATGTTCAGGTTGACCATCAC |
| RORC | GTGGGGACAAGTCGTCTGG | AGTGCTGGCATCGGTTTCG |
| IFNγ | TCGGTAACTGACTTGAATGTCCA | TCGCTTCCCTGTTTTAGCTGC |
| T-bet | GGTTGCGGAGACATGCTGA | GTAGGCGTAGGCTCCAAGG |
| FOXP3 | GTGGCCCGGATGTGAGAAG | GGAGCCCTTGTCGGATGATG |
